# The state of heart disease in Sudan

**DOI:** 10.5830/CVJA-2010-054

**Published:** 2011-08

**Authors:** A Suliman

**Affiliations:** Department of Medicine, Faculty of Medicine, University of Khartoum, Khartoum, Sudan

**Keywords:** Sudan, prevalence, epidemiology, heart disease, pattern of heart disease, hypertensive heart disease, rheumatic heart disease, ischaemic heart disease, cardiomyopathy

## Abstract

**Abstract:**

Cardiovascular disease (CVD) is the leading cause of mortality worldwide and an important cause of disability. In Africa, the burden of CVD is increasing rapidly and it is now a public health concern. Epidemiological data on diseases is scarce and fragmented on the continent.

**Aim:**

To review available data on the epidemiology and pattern of heart disease in Sudan.

**Methods:**

Data were obtained from the Sudan Household Survey (SHHS) 2006, annual health statistical reports of the Sudan Federal Ministry of Health, the STEPS survey of chronic disease risk factors in Sudan/Khartoum, and journal publications.

**Results:**

The SHHS reported a prevalence of 2.5% for heart disease. Hypertensive heart disease (HHD), rheumatic heart disease (RHD), ischaemic heart disease (IHD) and cardiomyopathy constitute more than 80% of CVD in Sudan. Hypertension (HTN) had a prevalence of 20.1 and 20.4% in the SHHS and STEPS survey, respectively. There were poor control rates and a high prevalence of target-organ damage in the local studies. RHD prevalence data were available only for Khartoum state and the incidence has dropped from 3/1 000 people in the 1980s to 0.3% in 2003. There were no data on any other states. The coronary event rates in 1989 were 112/100 000 people, with a total mortality of 36/100 000. Prevalence rates of low physical activity, obesity, HTN, hypercholesterolaemia, diabetes and smoking were 86.8, 53.9, 23.6, 19.8, 19.2 and 12%, respectively, in the STEPS survey. Peripartum cardiomyopathy occurs at a rate of 1.5% of all deliveries. Congenital heart disease is prevalent in 0.2% of children.

**Conclusion:**

Heart diseases are an important cause of morbidity and mortality in Sudan. The tetrad of hypertension, RHD, IHD and cardiomyopathy constitute the bulk of CVD. Hypertension is prevalent, with poor control rates. A decline in rheumatic heart disease was seen in the capital state and no data were available on other parts of the country. No recent data on IHD were available. Peripartum cardiomyopathy and congenital heart disease occur at similar rates to those in other African countries.

## Abstract

Sudan is the largest country in Africa and the ninth largest in the world, with an area of about one million square miles. It has a population of more than 39 million people, comprising tribes that descended from African, Arab and Nubian origins.^1^,^2^ Sudan is geographically unique, lying in the north-east corner of the continent, extending from latitude 22° north to 3° south, with the northern part of the country in the Saharan belt and the central and southern parts in the sub-Saharan region.^1^

Worldwide, cardiovascular disease (CVD) is responsible for 30% of all deaths and 10% of DALYs (disability-adjusted life years).^3^ In Africa, the burden of cardiovascular disease is increasing rapidly and it is now a public health concern. It has a major socio-economic impact on individuals, families and societies in terms of healthcare costs, work absenteeism and national productivity. The most important cardiovascular diseases in the African region are those related to hypertension, atherosclerosis, cardiomyopathies and rheumatic heart disease.^4^,^5^

In Sudan, like many other less-developed countries, particularly in sub-Saharan Africa, epidemiological data on diseases are scarce and fragmented.^6^ Most of the data on disease burden for sub-Saharan Africa come from extrapolations, as in the Global Burden of Disease Study (GBDS),^7^ which relies on cause-of-death models and expert opinion.

In this article, we aim to review available epidemiological data on the burden of cardiovascular disease and its pattern in Sudan, as well as the prevalence of individual cardiac diseases, namely hypertensive, rheumatic and atherosclerotic heart diseases and their risk factors, cardiomyopathies, particularly peripartum and dilated cardiomyopathy, and congenital heart diseases.

We reviewed data from annual health statistical reports^8^ issued by the Federal Ministry of Health, the Sudan Household Survey (SHHS) of 2006,^9^ World Health Organisation STEPS (STEPwise approach to Surveillance) data,^10^ published data using Medline search, with the terms ‘cardiovascular disease Sudan’, ‘heart disease Sudan’, ‘hypertensive heart disease Sudan’ and ‘cardiomyopathy Sudan’, and unpublished data on relevant topics. These were compared to similar data from other countries in the continent and worldwide, as well as estimates from the World Health Organisation (WHO) and the Global Burden of Disease Study.^11^

## Burden of cardiovascular disease in Sudan

**Fig. 1.**
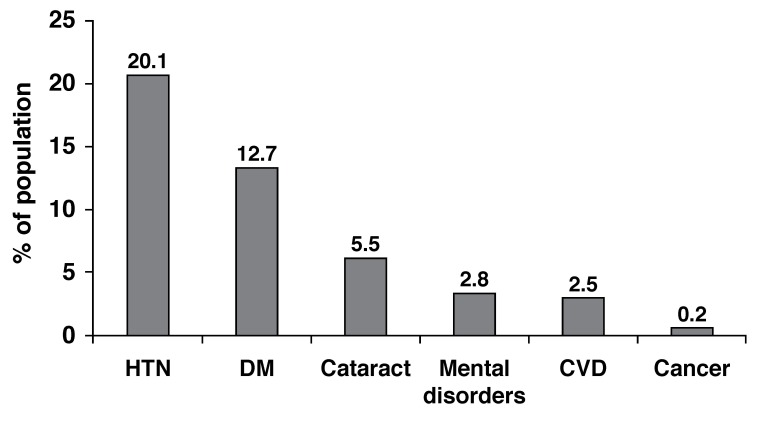
Prevalence of cardiovascular diseases, hypertension, diabetes mellitus, cancer, cataract and mental disorders in SHHS.^9^ CVD, cardiovascular disease; HTN, hypertension; DM, diabetes mellitus; SHHS, Sudan Household survey. Available at http://www.ssccse.org/blog/surveys.

The only epidemiological data on the prevalence of cardiovascular disease in the community come from the SHHS.^9^ This survey of 2006 was a questionnaire-based survey in all states of Sudan, organised by the Federal Ministry of Health and Ministry of Health of the Government of southern Sudan; 24 527 households and more than 55 000 Sudanese were surveyed. The self-reported prevalence of heart disease was 2.5%. This figure remains low compared to developed countries.^12^ Fig. 1 shows prevalence of cardiovascular disease compared to other non-communicable diseases in the SHHS.

**Fig. 2.**
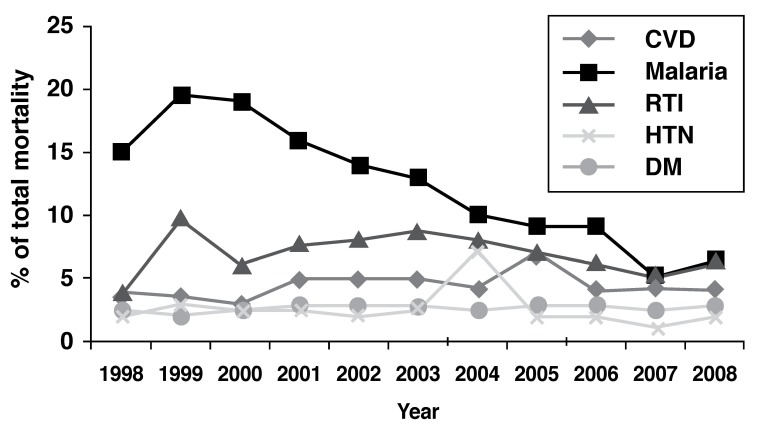
Hospital mortality of cardiovascular diseases, hypertension, diabetes mellitus, malaria and respiratory tract infections in Sudan 1998–2008. CVD, cardiovascular disease; HTN, hypertension; DM, diabetes mellitus; RTI, respiratory tract infection. Adapted from annual health statistical reports of the Federal Minsitry of Health.^10^ Available at http://fmoh.gov.sd/indexAr/php?id=6.

The Federal Ministry of Health issues an annual health statistical report that includes data on causes of hospital mortality. Over the past decade, cardiovascular disease has been consistently reported in the top 10 causes of hospital mortality, with malaria and acute respiratory infections as the first two causes. Fig. 2 demonstrates the contribution of cardiovascular disease, malaria, respiratory tract infections, diabetes mellitus (DM) and hypertension to hospital mortality in Sudan from 1998 to 2008. Such a significant contribution of cardiovascular disease to mortality is also seen in other African countries.^13^,^14^

## Pattern of heart disease in Sudan

In 1937, an analysis was made of 100 consecutive cases of heart disease admitted to Khartoum Hospital: 80 had cardiovascular syphilis, followed by rheumatic heart disease. There was no mention of ischaemic heart disease.

In 1961 the same author, Dr Halim, analysed 958 consecutive cardiac cases admitted to Khartoum Hospital. Hypertension and rheumatic heart disease (RHD) were the commonest two diagnoses at 44.4 and 25.4% of the sample, respectively. Ischaemic heart disease (IHD) was 12.6 % and syphilitic heart disease regressed to 6.0%.^15^ Table 1 summarises the result of that study.

**Table 1.** Analysis Of 958 Cardiac Cases Investigated In Khartoum During The Years 1957–1960^15^

*Cardiac disorder*	*Congenital*	*SA*	*HTN*	*IHD*	*PHD*	*EMF*	*RHD*	*Miscellaneous*
5 of total	3.7	6.0	44.4	12.6	2.0	3.2	25.4	2.7

SA, syphilitic aortitis; HTN, hypertension; IHD, ischaemic heart disease; PHD, pulmonary heart disease; EMF, endomyocardial fibrosis; RHD, rheumatic heart disease.

Two other similar studies investigating the pattern of heart disease were conducted in the 1980s and 1990s. The first study conducted by Khalil *et al*. was in a community hospital in Khartoum North Hospital and included 539 patients from 1980 to 1983.^16^ The other was performed in 1992 in Al Shaab Hospital, which is a tertiary referral hospital, by Kurdufani *et al*. and included 1 000 patients. Unfortunately, the latter study was unpublished and data were obtained directly from the author.

Table 2 summarises the main findings from these two studies. They show that the tetrad of hypertensive heart disease, rheumatic heart disease, ischaemic heart disease and cardiomyopathy are the main cardiovascular causes for hospital admission. These results are comparable to the recent Heart of Soweto study,^17^ which also showed that these four disease categories, together with pericardial disease, are the main cardiac causes for hospital presentation in Soweto, South Africa.

**Table 2.** Summary Of Recent Pattern Of Heart Disease Studies In Sudan^16^

*Study (hospital, year)*	*RHD (%)*	*HHD (%)*	*IHD (%)*	*Cardiomyopahty (%)*	*CHD (%)*	*Other (%)*
Khalil *et al*. (Khartoum North Hospital, 1980–1983)	26.5	33.7	17.8	4.8	3.9	13.3
Kurdufani *et al*. (AlShaab Hospital, 1992)	30.0	12.0	32.0	6.0	2.0	18.0

RHD, rheumatic heart disease; HHD, hypertensive heart disease; IHD ishaemic heart disease; CHD, congenital heart disease.

The contribution of HIV/AIDS to heart disease in Sudan, particularly cardiomyopathy and pericardial disease is unclear. However, this is expected to be less than what is seen in many parts of sub-Saharan Africa, as Sudan has one of the lowest HIV prevalence rates in this region. HIV prevalence rate for adults aged 15 to 49 in Sudan is 1.4% (1–2%),^18^ compared to 5.2% for sub-Saharan Africa in 2008.^19^

## Hypertensive heart disease

Hospital-based surveys in Sudan dating back from the middle of the last century have shown that hypertensive heart disease, particularly with its contribution to heart failure, is probably the commonest cause of cardiovascular disease.^15^,^16^ This can be explained by a number of factors, including high prevalence rates of hypertension, particularly in urban communities, poor control, and the larger contribution of hypertension to CVD in African patients. The SHHS and the STEPS survey have reported prevalence rates of 20.1 and 20.4%, respectively for hypertension. Also, the control of blood pressure in hypertensive patients seems to be poor in Sudan.

Two outpatient surveys have been identified that assessed this issue, both using cut-off points of < 140/90 mmHg for good control. The first was conducted in eastern Sudan and showed control rates of 19.4%,^20^ and the other was in Khartoum state and showed a control rate of only 28.1%.^21^ Both surveys showed similar compliance rates with prescribed medication, of 59.6 and 59.4%, respectively. The commonest cause of non-compliance was inability to purchase medication.

The prevalence of target-organ damage, mainly cardiac and renal, in outpatient clinics in Sudan is relatively high, with at least one-fifth of the hypertensive population showing evidence of target-organ damage.^22^,^23^ Hypertension itself, as shown in the INTERHEART study, is a strong contributor to the hazards of CVD in black Africans, with an odds ratio (OR) of 7.0 versus 2.3–3.9 in other ethnic groups (*p* = 0.0002).^24^

This situation is not unique to Sudan and is seen throughout the continent, where hypertension is the leading cardiovascular disease and cause of heart failure.^17^,^25^,^26^ However, this condition is treatable and to some extent preventable.^4^

## Prevalence of rheumatic heart diseases

The highest prevalence of RHD is in sub-Saharan Africa, with a prevalence of 5.7 per 1 000 people, compared with 1.8 per 1 000 in North Africa, and 0.3 per 1 000 in economically developed countries with established market economies.^27^

The epidemiological data on rheumatic valvular heart disease in Sudan come from the WHO Global Rheumatic Fever/Rheumatic Heart Disease Prevention Program in Sudan. This project had two phases. Phase I was from 1986 to 1989, where more than 13 000 schoolchildren aged five to 15 years in Sahafa town were screened clinically. Sahafa was chosen because it had well-marked houses and streets and most of its inhabitants were low- and middle-income families that had moved to the capital from all districts of the country following the drought in the early 1980s. Prevalence of rheumatic heart disease in Sudan was found to be 3/1 000 population,^28^ as quoted by the World Health Organisation.^29^

Phase II of the programme was conducted from 1994 to 2003 in the state of Khartoum; 1 095 000 schoolchildren were screened in this phase. Prevalence was found to be 0.3/1 000 after implementation of a primary and secondary prevention programme. These data were presented by Dr N Kordofani at the 2006 World Congress of Cardiology in Barcelona, Spain. There are no data on other parts of the country. The 10-fold drop in RHD prevalence over less than two decades, seen in the capital state of Khartoum, due to screening and prevention is not expected in other states where no formal programme exists.

However, the recent work by Marijon *et al.*, which demonstrated that the prevalence of RHD as detected by echocardiographic screening is 10 times that of clinical screening.^30^ This raises a number of concerns for Sudan and the continent regarding the true prevalence of RHD and the feasibility and cost-effectiveness of echocardiographic screening.

## Atherosclerotic heart diseases and risk factors

Ischaemic heart disease is the leading cause of death worldwide, both in high-income and low- and middle-income countries, except in sub-Saharan Africa where HIV/AIDS and infectious disease, mainly malaria, are the major causes of death. It is also responsible for 10% of DALYs lost in low- and middle-income countries and 18% in high-income countries.^31^,^32^ The incidence of ischaemic heart disease in Africa has risen greatly in the last decade and it has been estimated that it ranked eighth in the leading causes of death, and number one in those over 60 years of age.^33^-^35^

There has been only one population-based study in Sudan, conducted by Khalil *et al*., which addressed the issue of coronary event rates.^36^ All coronary events occurring in Khartoum, capital of Sudan, were registered during the calendar year 1989 using the diagnostic and classification criteria of the World Health Organisation Monitoring of Trends and Determinants in Cardiovascular Disease (MONICA) project.^37^ The annual (1989) coronary event rate was 112/100 000 with a total mortality of 36/100 000. The highest event rate of 364/100 000 occurred in men aged 45 to 64 years. The event rates recorded in this study were low compared to most other MONICA centres, e.g. Spain (Catalonia) 187/100 000, Australia (Newcastle) 561/100 000 and Canada (Halifax) 605/100 000.^38^

However, these data are two decades old and it is believed that during this time, many third-world countries, and Sudan is no exception, have entered a period of epidemiological transition. Greater urbanisation and economic development has led to a shift in the major causes of death and disability, from infectious diseases to chronic non-communicable diseases such as cardiovascular disease and cancer.^39^

The WHO 2002 estimates for IHD in Sudan,^40^ based on the Global Burden of Disease study,^26^ are an age-adjusted mortality rate of 205/100 000 and an age-adjusted DALYs of 1185/100 000 population. Such estimates need to be validated by local surveys. Table 3 presents age-standardised mortality rates for all-cause, non-communicable diseases, cardiovascular diseases, IHD and DALYs for IHD for Sudan and other African states from different regions of the continent.

**Table 3.** Shows Mortality Rates For All-Cause, CVD And IHD And Years Of Life Lost In Sudan And Selected African Countries From Different Regions Of The Continent

*Country*	*Age-standardised all-cause mortality rates per 100 000*	*Age-standardised mortality rate for NCD per 100 000*	*Age-standardised mortality rate for CVD per 100 000*	*Age-standardised mortality rate for IHD per 100 000*	*Age-standardised DALYs for IHD per 100 000*
Sudan	1495	902	499	204	1185
Egypt	1132	958	560	273	1781
Eritrea	1584	762	398	124	679
Ghana	1510	786	404	128	726
South Africa	2011	808	406	124	758

Source: World Health Organisation. Death and DALY estimates by cause, 2002. Available at http://www.who.int/entity/healthinfo/statistics/bodgbddeathdalyestimates.xls (accessed 06 December 2009). NCD, non-communicable disease; CVD, cardiovascular disease; IHD, ischaemic heart disease; DALYs, disability-adjusted life years.

**Fig. 3.**
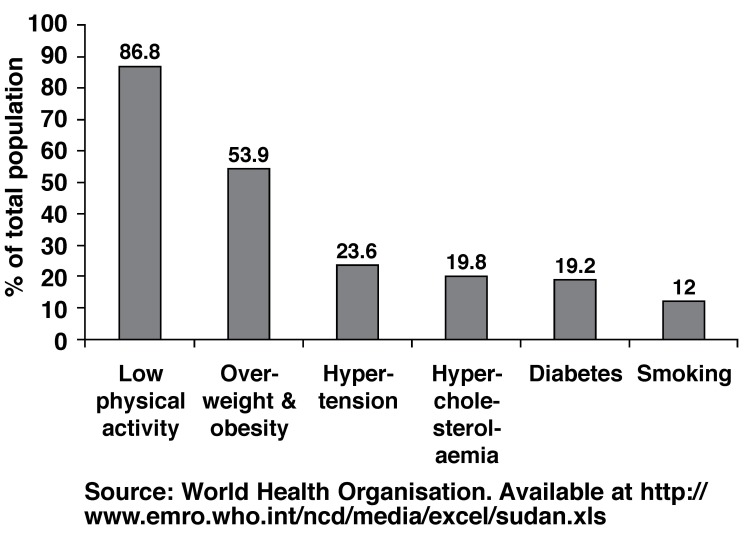
Results of STEPS survey in Sudan 2005/2006.

Several surveys and studies into the prevalence of risk factors of atherosclerotic disease in Sudan were conducted in recent years. The SHHS showed a self-reported prevalence of hypertension and DM of 20.4 and 12.7%, respectively. The STEPS survey^41^ of chronic risk factors for IHD, carried out in Khartoum state from December 2005 to January 2006 showed high prevalence rates of these risk factors. Fig. 3 illustrates prevalence rates for hypertension, DM, obesity, hypercholesterolaemia, smoking and physical inactivity in the STEPS survey.

The high prevalence of these risk factors is alarming. The INTERHEART study showed that the nine risk factors (smoking, diabetes, hypertension, increased ratio of apolipoprotein B to apoliporotein A-1, increased weight-to-hip ratio, low consumption of fruits and vegetables, low physical activity, no alcohol intake and psychosocial stress) provided population-attributable risk (PAR) for developing a first-time myocardial infarction of 90.4% worldwide^42^ and 97.4% in Africa.^24^ Of these risk factors, only five (current/former tobacco smoking, self-reported diabetes and hypertension, abdominal obesity measured as waist-to-hip ratio, and elevated ApoB/ApoA-1 ratio) accounted for 78.4% of the PAR worldwide^42^ and 89.2% in the African participants.^24^

## Cardiomyopathies

## Idiopathic dilated cardiomyopathy (DCM)

Idiopathic DCM is a major cause of heart failure in Africa.^43^,^44^ However, there are no population-based data on the burden of the disease in Africa and most data come from hospital-based surveys.

Earlier hospital-based surveys show that cardiomyopathies constitute 4 to 6% of all cardiac admissions.^16^ In our cardiology unit at the Al Shaab Teaching Hospital in Khartoum, where the National Cardiothoracic Centre is located, 12% of all admissions in 2009 were due to idiopathic DCM. Many clinicians in Sudan believe that DCM is becoming more prevalent. Lack of epidemiological data that support such assumptions hinder the recognition of this disease as a major health issue.

## Endomyocardial fibrosis (EMF)

No epidemiological data are available on the prevalence of EMF in Sudan. Limited data are available from hospital-based surveys. In adults it seems to be a rare cause of heart disease.^15^ In the paediatric population, it appears to be a more important cause. Ali reviewed all paediatric patients with cardiac disease admitted at the Children’s Hospital, Khartoum from September 2007 to September 2008 and identified six patients with EMF, constituting 18% of all children with cardiomyopathy.^45^

## Peripartum cardiomyopathy (PPCM)

The only data on the incidence of PPCM come from unpublished work by Kineish *et al*. All deliveries in Khartoum Teaching Hospital from 1975 to 1979 were screened. Any woman who developed heart failure during the last trimester or during puerperium was examined clinically, and evaluated by electrocardiogram and chest X-ray. If no identifiable cause of heart failure was found, patients were labelled as having PPCM. Thirteen patients were identified out of 8 605 deliveries, with an incidence of 1.5 in 1 000 deliveries. This is similar to the incidence in the sub-Saharan region, except possibly in the Zaria province in northern Nigeria, which has the highest reported incidence rate of one in 100 deliveries.^46^,^47^

## Congenital heart disease

The prevalence of congenital heart disease among schoolchildren aged five to 15 years was studied as part of phase 1 of the WHO Global Rheumatic Fever/Rheumatic Heart Disease Prevention Programme in Sudan. There were 27 cases of congenital heart disease found in a total of 13 322 children screened, giving a prevalence rate of 2.0 per 1 000 children. The rate is comparable to that of similar African countries but lower than European and North American rates.^49^-^53^

Among children admitted to hospital, congenital heart disease is the commonest cause of heart disease, followed by rheumatic heart disease and cardiomyopathy. Ventricular septal defect, atrial septal defect, tetralogy of Fallot, patent ductus arteriosus and pulmonary stenosis were the commonest diseases.^54^,^55^

## Conclusion

Heart disease is prevalent in Sudan, with at least 2.5% of the population affected, and it is one of the major causes of hospital mortality. The tetrad of hypertensive heart disease, ischaemic heart disease, rheumatic heart disease and cardiomyopathy constitute the bulk of heart disease.

Hypertension is prevalent, especially in urban communities, with poor control rates. Data on RHD are only available for the capital state of Khartoum, where a prevention programme succeeded in reducing prevalence 10-fold from 3/1 000 to 0.3/1 000 population. There are no recent epidemiological data on the prevalence of IHD. However, IHD risk factors are alarmingly prevalent in the community.

Prevalence of cardiomyopathies is not known, although it seems clinicians are recognising idiopathic dilated cardiomyopathy more frequently. EMF is rarely reported in adult patients in recent literature but is seen infrequently in paediatric population. Peripartum cardiomyopathy seems to occur at a similar incidence to that in other sub-Saharan countries. The prevalence rate of congenital heart disease is comparable to other African countries but lower than European and North American rates. Epidemiological data are scarce and fragmented.

The need for quality data cannot be overemphasised. However, there are enough existing data to warrant primary and secondary prevention programmes for risk factors for heart disease to go hand in hand with epidemiological surveys.
